# Mean field approximation for solving QUBO problems

**DOI:** 10.1371/journal.pone.0273709

**Published:** 2022-08-30

**Authors:** Máté Tibor Veszeli, Gábor Vattay

**Affiliations:** Department of Physics of Complex Systems, Eötvös Loránd University, Budapest, Hungary; Central State University & Ohio University, UNITED STATES

## Abstract

The Quadratic Unconstrained Binary Optimization (QUBO) problem is NP-hard. Some exact methods like the Branch-and-Bound algorithm are suitable for small problems. Some approximations like stochastic simulated annealing for discrete variables or mean-field annealing for continuous variables exist for larger ones, and quantum computers based on the quantum adiabatic annealing principle have also been developed. Here we show that the mean-field approximation of the quantum adiabatic annealing leads to equations similar to those of thermal mean-field annealing. However, a new type of sigmoid function replaces the thermal one. The new mean-field quantum adiabatic annealing can replicate the best-known cut values on some of the popular benchmark Maximum Cut problems.

## 1 Introduction

From solid state physics [1] to social phenomena [2] Ising models can describe a wide range of complex systems. Spin models are versatile because they are simple yet able to show complex phenomena, like emergence and phase transitions [3–5]. Spin models are also important in large, real-life optimization problems which can be distilled down to finding the global minimum of a high-dimensional, nonlinear function. Most of these tasks are NP-hard [6]. Small problems up to approximately a hundred nodes for a dense problem can be solved by exact methods like the Branch-and-Bound (B&B) [7], or Branch-and-Cut [8]. Larger problems can be solved approximately with intuitive methods like the tabu search [9, 10], semi-definite programming [11–13], stochastic simulated annealing (SSA) [14–16] and quantum annealing [17–19]. Quantum annealing is also the key idea behind adiabatic quantum computers [20, 21] such as the D-Wave system [22–25]. To reduce the computational costs of stochastic methods, the mean-field annealing (MFA) [26] has been proposed. In MFA, the dynamics of spins are replaced with the evolution of their average values. As the temperature decreases, the MFA algorithm updates these averages based on their values at the previous temperature. Computation using the means reaches equilibrium faster than the corresponding stochastic dynamics, and MFA relaxes to a solution much faster than SSA, leading to an overall decrease in computational effort. Such methods have become popular lately, and special devices like coherent Ising machines [27, 28] have been developed.

In this paper, we develop the mean-field version of the quantum annealing [17] method and apply it to the maximum cut problem, which belongs to the larger set of Quadratic Unconstrained Binary Optimization (QUBO) problems. The mean-field quantum annealing introduced here is similar to [18, 19] in spirit. The novelty is the proposed quantum MFA is that the equations derived here are fully analogous to the thermal MFA equations, the tunneling rate of the quantum MFA corresponds to the inverse temperature, and the sigmoid theta function is replaced with a new and different sigmoid function. The new sigmoid function improves the performance of the quantum MFA, which is in line with recent result [29] emphasizing the importance of the shape of the sigmoid function.

The structure of this paper is the following. In section 2 we summarize the thermal mean-field annealing method for the Ising problem. Then in section 3 we present the quantum annealing problem and its proposed novel mean-field solution. In section 4 we test the new method on the maximum cut problem benchmark G-sets [30], and finally, in section 5 we compare the new method with SSA and B&B.

## 2 Thermal mean-field annealing

The variational principle of statistical physics is a powerful tool for examining interacting systems at finite temperatures. The most straightforward version of this principle is the mean-field approximation. This approximation works best when the number of interactions per spin is large. In real-life optimization problems, the number of nodes is usually not as large as in physics, typically just tens of thousands, and usually, they have no symmetry. The statistical physical derivation of the mean-field annealing starts from the expression of the free energy

$$\begin{array}{c} F\left(T\right)=-T\;\text{ln}\sum_{n}e^{-E_{n}/T}, \end{array}$$ (1)

at finite temperature, *T*, where the sum goes over all the system states, and *E*_*n*_ is the energy. The probability of finding the system at state *n* is proportional to the Boltzmann weight ${\text{e}}^{-E_{n}/T}$, therefore, the free energy coincides with the ground state energy at zero temperature. The problem is that even if we know all the *E*_*n*_ energies, apart from the most straightforward cases, we cannot evaluate the summation due to the exponentially large number of terms. The variational principle states that the exact free energy is always smaller or equal to variational free energy

$$\begin{array}{c} F^{V}\left(T\right)=\sum_{n}P_{n}E_{n}+T\sum_{n}P_{n}\;\text{ln}\left(P_{n}\right), \end{array}$$ (2)

where *P*_*n*_ is an arbitrary probability distribution. The closer the probabilities *P*_*n*_ are to the real ones, the lower *F*^*V*^(*T*) will be. In practice, the distribution can’t be too complicated since we have to calculate the variational free energy analytically, avoiding exponential summations. The typical strategy is to consider a class of probability distributions *P*_*n*_(**m**) parametrized by some set of parameters **m**, then calculate *F*^*V*^ (**m**, *T*) and finally find the minimum m*. This solution is temperature dependent and we will refer to it as m*(*T*). The variational free energy is then *F*^*V*^(*T*) = *F*^*V*^ (m*(*T*), *T*).

The Ising model consist of interacting spins: **S** = (*S*_1_, *S*_2_ … *S*_*N*_), with components *S*_*i*_ ∈ {±1}. The model is defined by its energy:

$$\begin{array}{c} E\left(S\right)=-\frac{1}{2}\sum_{ij}J_{ij}S_{i}S_{j}-\sum_{i}h_{i}S_{j}, \end{array}$$ (3)

where *J*_*ij*_ is the interaction between spin *i* and *j*, and *h*_*i*_ is the external magnetic field. In the mean field approximation, we assume that a variational distribution factorizes. In the case of spin systems

$$\begin{array}{c} P^{MF}\left(S;m\right)=\prod_{i=1}^{N}P_{i}\left(S_{i};m_{i}\right)=\prod_{i}\frac{1+m_{i}S_{i}}{2}. \end{array}$$ (4)

This distribution is normalized and the expectation values of the spins are the parameters 〈*S*_*i*_〉 = *m*_*i*_. The variational free energy is

$$\begin{array}{ll} F^{MF}(m,T)= & -\frac{1}{2}\sum_{ij}J_{ij}m_{i}m_{j}-\sum_{i}h_{i}m_{j}\\ & +T\sum_{i}[\frac{1+m_{i}}{2}\;\text{ln}\left(\frac{1+m_{i}}{2}\right)+\frac{1-m_{i}}{2}\;\text{ln}\left(\frac{1-m_{i}}{2}\right)]. \end{array}$$ (5)

We have to find its minimum, so its derivative has to be zero

$$\begin{array}{c} \frac{\partial F^{MF}}{\partial m_{i}}=-\sum_{j}J_{ij}m_{j}-h_{i}+T\frac{1}{2}\text{ln}(\frac{1+m_{i}}{1-m_{i}})=0 \end{array}$$ (6)

and the second derivative, the Hessian should be positive definite

$$\begin{array}{c} \frac{\partial^{2}F^{\text{MF}}}{\partial m_{i}\partial m_{j}}=-J_{ij}+\frac{T\delta_{ij}}{1-m_{i}^{2}}≻0. \end{array}$$ (7)

We can rewrite the implicit Eq (6) as a self-consistent equation:

$$\begin{array}{c} m_{i}=\text{tanh}[\frac{h_{i}+\sum_{j}\;J_{ij}m_{j}}{T}]. \end{array}$$ (8)

If **m**(*T*) is known for a given temperature *T*, we can determine it for a slightly lower temperature **m**(*T* − Δ*T*) by using the self-consistent equation iteratively as it was proposed in [26]. This method requires fewer function evaluations than SSA. Above some temperature *T* > *T*_0_ the iterative equation

$$\begin{array}{c} m_{i}\left(T-\Delta T\right)=\text{tanh}[\frac{h_{i}+\sum_{j}\;J_{ij}m_{j}\left(T\right)}{T-\Delta T}] \end{array}$$ (9)

converges towards a unique high temperature solution *m*_*i*_ ≈ *h*_*i*_/*T*. There are more solutions at lower temperatures. At *T*_0_, a bifurcation occurs, and the iterations after that follow one of the new post-bifurcation solutions. Later on at successively lower temperatures *T*_1_ > *T*_2_ > *T*_3_… further bifurcations occur, and the iteration follows one of the new solutions. The bifurcation temperatures *T*_*n*_ are those points, where one of the eigenvalues and consequently the determinant of the Hessian (7) becomes zero

$$\begin{array}{c} \text{det}(-J_{ij}+\frac{T_{n}\delta_{ij}}{1-m_{i}^{2}\left(T_{n}\right)})=0 \end{array}$$ (10)

at the solution *m*_*i*_(*T*_*n*_) of the self-consistent Eq (8). Due to the nature of the solution, the mean-field annealing is also called a bifurcation machine. The procedure described above leads to one of the local minima of free energy. In general, there is no guarantee that the method leads to the absolute minimum, but decreasing the size of cooling steps Δ*T* and increasing the overall running time in parallel, improves the chances of finding deeper minima [31].

## 3 Quantum mean field annealing

Instead of the free energy and temperature, we can use quantum mechanics and the adiabatic theorem to determine the system’s ground state. The adiabatic theorem asserts that if a quantum system is initially at the ground state and the corresponding time-dependent Hamiltonian operator changes sufficiently slowly, there is a gap between the ground state energy and the rest of the spectrum. The system remains at the instantaneous ground state [32]. An application of this theorem is to find the ground state of a complicated Hamiltonian. The ground state of the initial Hamiltonian *H*_i_ should be easy to prepare, and the final operator *H*_f_ is the one whose ground state we are interested in. In that case, we can compose the Hamilton operator of the adiabatic problem

$$\begin{array}{c} H\left(t\right)=(1-s(t))H_{\text{i}}+s\left(t\right)H_{\text{f}}, \end{array}$$ (11)

where *s*(*t*) is a continuous, monotonic function of time, with *s*(0) = 0 and *s*(*T*_A_) = 1. The time *T*_A_ is the annealing time, which must be large. The simplest choice for this is *s*(*t*) = *t*/*T*_A_. To solve the Ising problem with the adiabatic method we have to use the quantum version of the Ising model, as the final Hamilton operator

$$\begin{array}{c} H_{\text{f}}=-\frac{1}{2}\sum_{ij}J_{ij}\sigma_{i}^{z}\sigma_{j}^{z}-\sum_{i}h_{i}\sigma_{i}^{z}, \end{array}$$ (12)

where *σ*^*z*^ is the Pauli z-matrix. For the initial Hamilton operator the standard choice is the transverse magnetic field term

$$\begin{array}{c} H_{\text{i}}=-\Delta \sum_{i}\sigma_{i}^{x}, \end{array}$$ (13)

where Δ is the strength of the external field. This transverse term plays similar role to the entropy term in Eq (2) and it induces tunneling between the minima of the Ising Hamiltonian. The initial ground state is

$$\begin{array}{c} |\Psi_{0}\rangle =\overset{N}{\underset{i=1}{⊗}}\frac{\left|↑\rangle +|↓\right\rangle }{\sqrt{2}}, \end{array}$$ (14)

which is a product state, and after the annealing the final state is the ground state of Eq (12) and from that we can determine the minimal energy spin configuration of Eq (3). For a given *H*(*t*) the state of the system is governed by the Schrödinger equation.

$$\begin{array}{c} i\frac{d}{dt}\left|\Psi \left(t\right)\right\rangle =H\left(t\right)|\Psi \left(t\right)\rangle . \end{array}$$ (15)

Initially the system is at ground state: |Ψ(*t* = 0)〉 = |Ψ_0_〉, If the annealing time *T*_A_ is sufficiently large, then the evolving wave function reaches the ground state of the Ising model.

To develop an MFA to the quantum problem we introduce the energy functional

$$\begin{array}{c} E[|\Psi (s)\rangle ]=\langle \Psi (s)|H(s)|\Psi (s)\rangle . \end{array}$$ (16)

At *s* = 0, we set the initial wave function to the initial state |Ψ(0)〉 = |Ψ_0_〉 and for *s* > 0, we want to keep it in the minimal energy state. In the mean-field approximation, just like in the thermal case, we assume that the spins are independent and the trial state vector is a product

$$\begin{array}{c} \left|\Phi \left(s\right)\right\rangle =\overset{N}{\underset{i=1}{⊗}}[c_{i↓}\left(s\right)|↓\rangle +c_{i↑}\left(s\right)|↑\rangle ] \end{array}$$ (17)

where the amplitudes are constrained by |*c*_*i*↓_(*s*)|^2^ + |*c*_*i*↑_(*s*)|^2^ = 1. The expectation values of the Pauli operators can be expressed with the amplitudes as

$$\begin{array}{l} m_{i}^{z}=\langle \Phi |\sigma_{i}^{z}|\Phi \rangle =|c_{i↑}{|}^{2}-|c_{i↓}{|}^{2},\\ m_{i}^{x}=\langle \Phi |\sigma_{i}^{x}|\Phi \rangle =c_{i↑}^{*}c_{i↓}+c_{i↓}^{*}c_{i↑},\\ m_{i}^{y}=\langle \Phi |\sigma_{i}^{y}|\Phi \rangle =-ic_{i↑}^{*}c_{i↓}+ic_{i↓}^{*}c_{i↑}, \end{array}$$ (18)

where $m_{i}^{x}$, $m_{i}^{y}$ and $m_{i}^{z}$ are real. They satisfy ${\left(m_{i}^{x}\right)}^{2}+{\left(m_{i}^{y}\right)}^{2}+{\left(m_{i}^{z}\right)}^{2}=1$. It is easy to show that *c*_*i*↑_ and *c*_*i*↓_ can be chosen to be real, and in that case $m_{i}^{y}\left(s\right)=0$. In terms of the spin expectation values the energy functional is

$$E[m^{z}(s)]=s\left[-\frac{1}{2}\sum_{ij}J_{ij}m_{i}^{z}m_{j}^{z}-\sum_{i}h_{i}m_{i}^{z}\right]-(1-s)\Delta \sum_{i}\sqrt{1-{\left(m_{i}^{z}\right)}^{2}},$$ (19)

where we used $m_{i}^{x}=\sqrt{1-{\left(m_{i}^{z}\right)}^{2}}$, since $m_{i}^{x}>0$ during the minimization process. The parameter *s* plays the role of the temperature. The energy functional is minimal where the derivatives in terms of the expectation values vanish

$$\begin{array}{c} \frac{\partial E}{\partial m_{i}^{z}}=-s\sum_{j}J_{ij}m_{j}^{z}-h_{i}+\left(1-s\right)\Delta \frac{m_{i}^{z}}{\sqrt{1-{\left(m_{i}^{z}\right)}^{2}}}=0, \end{array}$$ (20)

and the Hessian is positive definite

$$\begin{array}{c} \frac{\partial^{2}E}{\partial m_{i}^{z}\partial m_{j}^{z}}=-sJ_{ij}+\left(1-s\right)\Delta \frac{\delta_{ij}}{{\left(1-{\left(m_{i}^{z}\right)}^{2}\right)}^{3/2}}≻0. \end{array}$$ (21)

**Fig 1:**
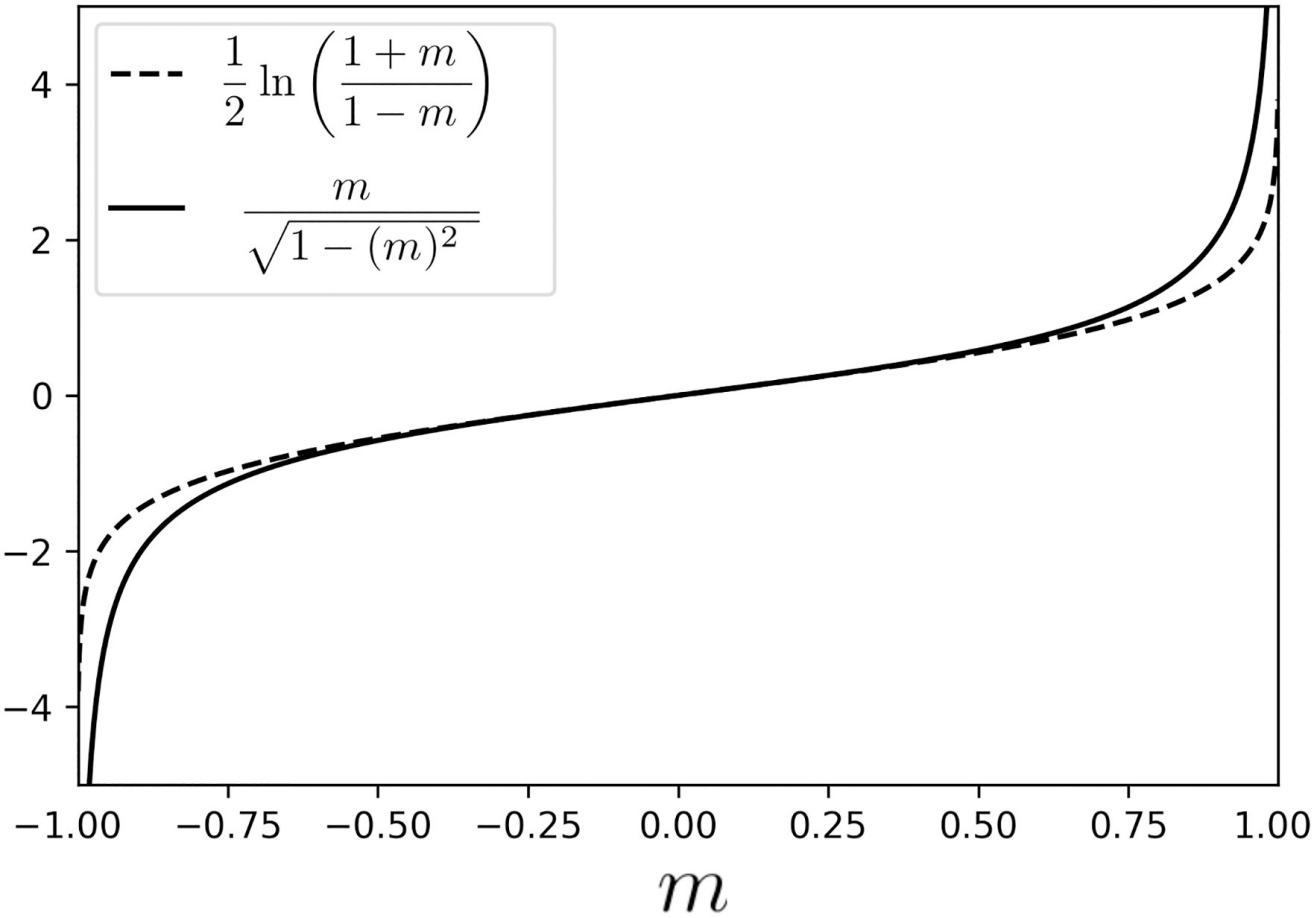
Comparison of the second term in the equations of the thermal (6) and the quantum (20) annealing.

The first part of Eq (20) is the same as in (6). The difference is in the second term, as it is seen in Fig 1. Now, we can rewrite the implicit Eq (20) as a self-consistent equation

$$\begin{array}{c} m_{i}=\sigma [\frac{h_{i}+\sum_{j}J_{ij}m_{j}}{\Gamma }], \end{array}$$ (22)

where the tunneling rate is Γ = Δ(1 − *s*)/*s* and we dropped the superscript $m_{i}=m_{i}^{z}$. The new sigmoid function is

$$\begin{array}{c} \sigma \left(x\right)=\frac{x}{\sqrt{1+x^{2}}}. \end{array}$$ (23)

This new equation is the main result of the paper. It is the equivalent of the thermal self-consistent Eq (8), where the tunneling rate Γ is the analog of of the temperature *T* and *σ*(*x*) replaces tanh(*x*).

The iterative solution of the new equation is similar to the thermal case. Given *m*_*i*_(*s* = 0) = 0 we would like to determine *m*_*i*_(*s* = 1). The **m**(*s*) vector is minimizing the energy functional *E*(**m**(*s*)), where we assume that the *s* ↦ **m**(*s*) trajectory is continuous. Without the external magnetic field the Ising model has a $Z_{2}$ symmetry. That means the $m_{i}^{z}=0$ is a solution to Eq (20), and it is a minimum as long as the smallest eigenvalue of the Hessian is above zero. For the parameter *s* this means

$$\begin{array}{c} s<\frac{\Delta }{\lambda_{\text{max}}\left(J\right)+\Delta } \end{array}$$ (24)

where λ_max_(**J**) is the largest eigenvalue of the matrix *J*_*ij*_. We can set Δ to 1 and rescale *J*_*ij*_ so that its largest eigenvalue is also 1. Now the trivial solution holds until *s* reaches 0.5. That means it is sufficient to start the simulation from *s* = 0.5. Once *m*_*i*_(*s* = 1) is known in the simulation, we round it to either 1 or -1. This gives us the **S** spin configuration. Since the derivative of *E*(**m**^*z*^) diverges as some *m*_*i*_ approaches ±1 it is advantageous to use a different parametrization *m*_*i*_(*ϑ*_*i*_) = cos(*ϑ*_*i*_), $m_{i}^{x}\left(\vartheta_{i}\right)=\sqrt{1-m_{i}^{2}}=\text{sin}\left(\vartheta_{i}\right)$ and then the derivative ∂*E*/∂*ϑ*_*i*_ = *s*(∑_*j*_ *J*_*ij*_ sin(*ϑ*_*i*_) cos(*ϑ*_*j*_) + *h*_*i*_ sin(*ϑ*_*i*_)) + (1 − *s*)(−Δ) cos(*ϑ*_*i*_) is regular for all *ϑ*_*i*_.

## 4 Benchmarking

As it was summarized by Lucas [33], many NP hard problems can be formulated with the Ising model. One of them is the QUBO problem, where we have to find the binary variables *x*_*i*_ ∈ {0, 1}, minimizing the quadratic functional argmin{*q*(**x**)}, where *q*(**x**) = ∑_*ij*_ *Q*_*ij*_ *x*_*i*_ *x*_*j*_, and **Q** is a symmetric matrix. Replacing the binary variables with spin variables *x*_*i*_ = (1 + *S*_*i*_)/2 leads to the Ising problem with parameters $J_{ij}=-\frac{1}{2}Q_{ij}$ and $h_{i}=-\frac{1}{2}\sum_{j}Q_{ij}$. The QUBO problem is then equivalent to finding the ground state of this Ising model. The NP-hard Maximum Cut problem [34] is often used as a benchmark for optimization algorithms. The task is to partition an undirected graph G=(V,E) into two subsets (S,V\S) such that the number of edges between these subsets is as large as possible. If the graph is defined via its adjacency matrix, where *A*_*ij*_ is one, if *i* and *j* are connected and zero otherwise, then the corresponding cut value is

$$\begin{array}{c} \text{CV}=\sum_{\{i,j\}\in E,i\in S,j\in V\backslash S}A_{ij}=\sum_{\underset{\left(i<j\right)}{i,j}}A_{ij}\frac{1-S_{i}S_{j}}{2}. \end{array}$$ (25)

In (25) *S*_*i*_ = +1 means the spin *i* is in the subset S, and *S*_*i*_ = −1 means it’s in V\S. Maximizing the cut value is equivalent to minimizing the Ising energy, where *J*_*ij*_ = −*A*_*ij*_ and *h*_*i*_ = 0.

**Fig 2:**
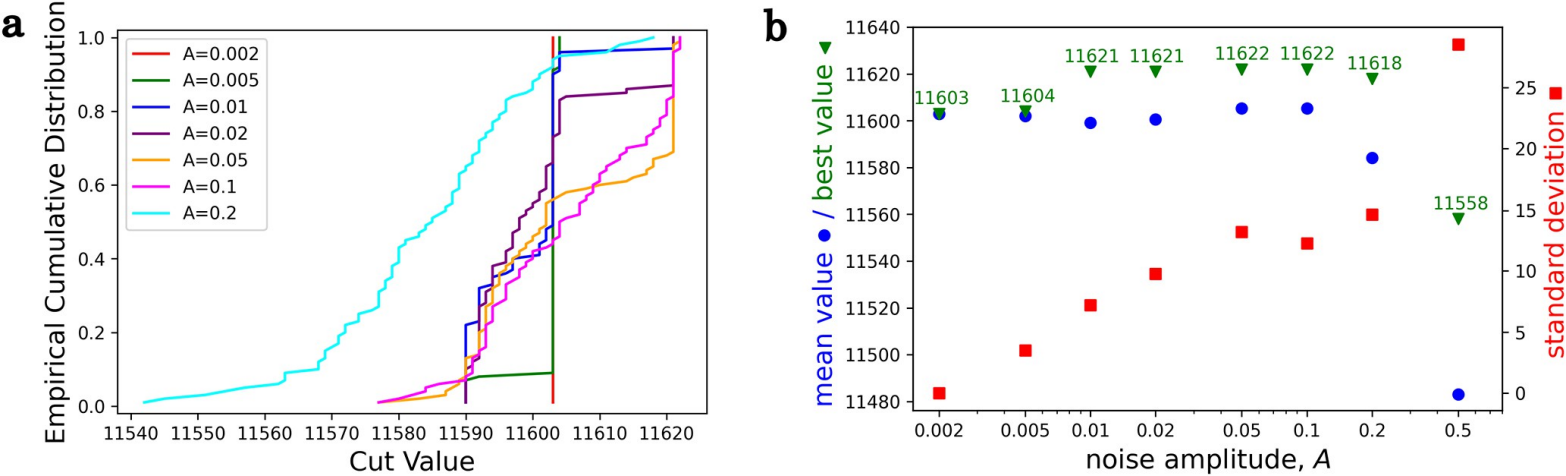
a, Empirical Cumulative Distribution of the Cut Values of G3 for different external magnetic field amplitudes. b, Mean value, best value and standard deviation of the Cut Values of G3 for different external magnetic field amplitudes.

During the simulation, the $Z_{2}$ symmetry is disadvantageous because, without the external field, the system remains in the $m_{i}^{z}=0$ solution forever. Adding some small random external magnetic field, *h*_*i*_ breaks this symmetry. We can use this field as noise and run the simulation repeatedly for many random realizations. One such distribution is shown in Fig 2a for the set G3 from G-set [30], which is one of the favorite problems to benchmark the methods for solving the Maximum Cut problem. This is a random graph with 800 nodes and 19176 links. The *h*_*i*_ components are randomly generated uniformly from (−*A*, *A*), where *A* is the amplitude, and one hundred trials were generated for all amplitudes. The mean values, the best values, and standard deviations are shown in Fig 2b. If the amplitude is small, we have a high average CV with a small deviation, on the other hand, if the amplitude is large, then the average CV is small, and the deviation is high. An optimal amplitude range exists, where the average is still large enough as well as the deviation, resulting in the largest best value from the sample. In the G3, this is around *A* = 0.05 − 0.1, where the best-known cat value 11622 has been achieved.

We tested our algorithm with other Max-Cut problems from the G-set. We focused on the smaller ones, i.e., the largest was the G22 graph with 2000 nodes. The results are summarized in Table 1. The last column shows the best values we could find in the literature [9, 35–39]. In most cases, our best results are the same as the best known; in the rest, it is close.

**Table 1:** G-set benchmarks. QMFA: Our results with the quantum MFA method. Best: Best-known results.

	G1	G2	G3	G4	G5	G6	G7	G8	G9	G10	G11	G12	G22
QMFA	11624	11620	11622	11646	11631	2178	2006	2005	2050	1999	560	554	13353
Best	11624	11620	11622	11646	11631	2178	2006	2005	2054	2000	564	556	13359

## 5 Comparison of the QMFA with SSA and B&B

Two widely used algorithms for discrete optimization problems are the stochastic simulated annealing (SSA), based on the Monte Carlo method, and the Branch-and-Bound method (B&B) [40]. The former is a metaheuristic algorithm, just like the QMFA, but has different hyperparameters. The latter is an exact algorithm where the tests were terminated after a fixed time due to the prior knowledge of its exponential time complexity. These two algorithms are used to compare the performance of the QMFA.

The pseudocode of QMFA is the following:

**algorithm**: Mean Field Annealing:

**preprocess**: $\underline{\underline{J}}\;≔\;\underline{\underline{J}}/\lambda_{\text{max}}\left(\underline{\underline{J}}\right)$, Δ ≔ 1

**input**: $E(\underline{\vartheta };\underline{h},s)$, $\partial_{\underline{\vartheta }}E\left(\underline{\vartheta };\underline{h},s\right)$, $\partial_{\underline{\vartheta }}\partial_{\underline{\vartheta }}E\left(\underline{\vartheta };\underline{h},s\right)$, *A*, *n*

**output**: $\underline{S}$

$\underline{\vartheta }\;≔\;\pi /2$

$\underline{h}$ ≔ random numbers between −*A* and *A*

**for** *k* ≔ 0 **to** *n*

 s ≔ 1/2 + k/(2n)

$\underline{\vartheta }\;≔\;$ local minimum of $E(\underline{\vartheta };\underline{h},s)$ using $\partial_{\underline{\vartheta }}E\left(\underline{\vartheta };\underline{h},s\right)$ and $\partial_{\underline{\vartheta }}\partial_{\underline{\vartheta }}E\left(\underline{\vartheta };\underline{h},s\right)$ with the initial value $\underline{\vartheta }$

$\underline{m}\;≔\;\text{cos}\left(\underline{\vartheta }\right)$

$\underline{S}$ ≔ (-1 **if** *m*_*i*_ < 0 **else** 1 **for** *m*_*i*_ **in** $\underline{m}$)

**return** $\underline{S}$

**Fig 3:**
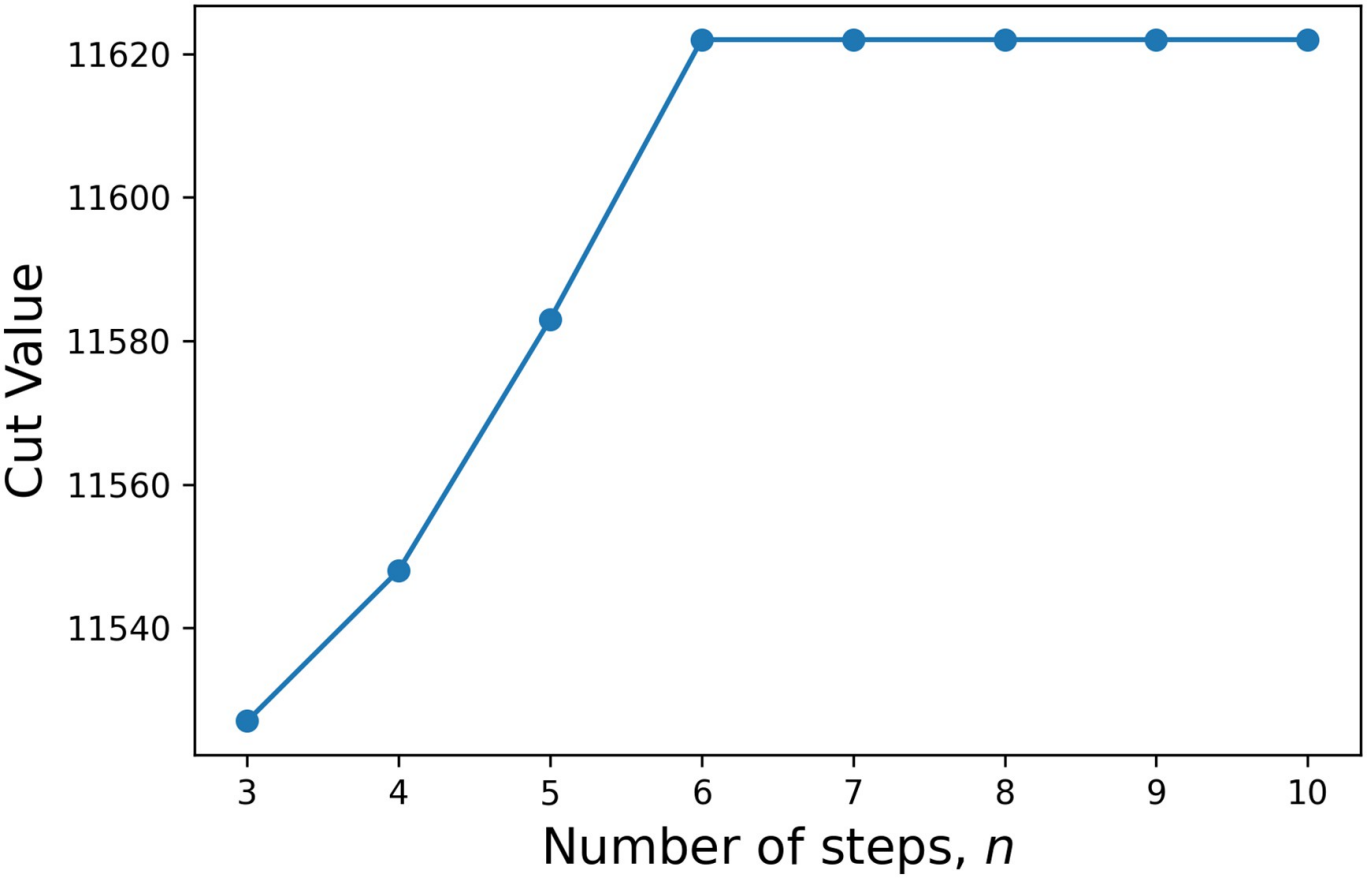
Convergence of the Cut Value of QFMA as a function of steps for the G1 problem.

We have used the Newton-conjugate-gradient method from the SciPy library for finding the local minimum. In this QMFA algorithm the noise amplitude *A* and the number of steps *n* are the hyperparameters, and once the vector $\underline{S}$ is known, the Cut Value is $CV=\frac{1}{4}\sum_{ij}J_{ij}\left(S_{i}S_{j}-1\right)$, with the original matrix $\underline{\underline{J}}$. Fig 3 shows one instance from G1 how the Cut Value converges as *n* increases. With *n* = 20, we can be sure that we have reached the final Cut Value. One run with *n* = 20 takes *t*_QMFA_ = (0.93 ± 0.18) s. Unfortunately, not every $\underline{h}$ noise gives the best Cut Value therefore, we have to run the algorithm multiple times. As shown in Fig 2 only a well-tuned noise amplitude can achieve a good result. For G1, the *A* = 0.1 is a good choice, but if we do not know this hyperparameter, we have to explore this parameter space as we did in Section 4. After we have determined *n* and *A*, we also need the success rate, i.e., the probability of finding the best-known Cut Value. For the G1 problem, out of 1000 trials, 61 were successful, so a rough estimation for the time needed to find the best value if the hyperparameters are known is 0.93 s ⋅ (1000/61) = 15.24 s.

In the case of SSA we need an annealing schedule [41], which tells us how the temperature decreases. We have chosen the geometrical cooling schedule, where *T*_*k*+ 1_ = *T*_*k*_ *α*, with *α* ∈ (0, 1). The pseudocode of the SSA is

**algorithm**: Stochastic Simulated Annealing

**input**: $E_{\underline{S}}$, *T*_init_, *α*, *n*

**output**: $\underline{S}$

$\underline{S}:=$ random vector with ±1 elements

**for** *k* ≔ 0 **to** *n*

 *i* ≔ random integer from [1, *N*]

 Δ*E*_*i*_ ≔ 2∑_*j*_ *J*_*ij*_ *S*_*i*_ *S*_*j*_

 *T* ≔ *T*_init_ *α*^*k*^

 **if** random(0,1) < exp(−Δ*E*_*i*_/*T*):

  *S*_*i*_ ≔ −*S*_*i*_

**Fig 4:**
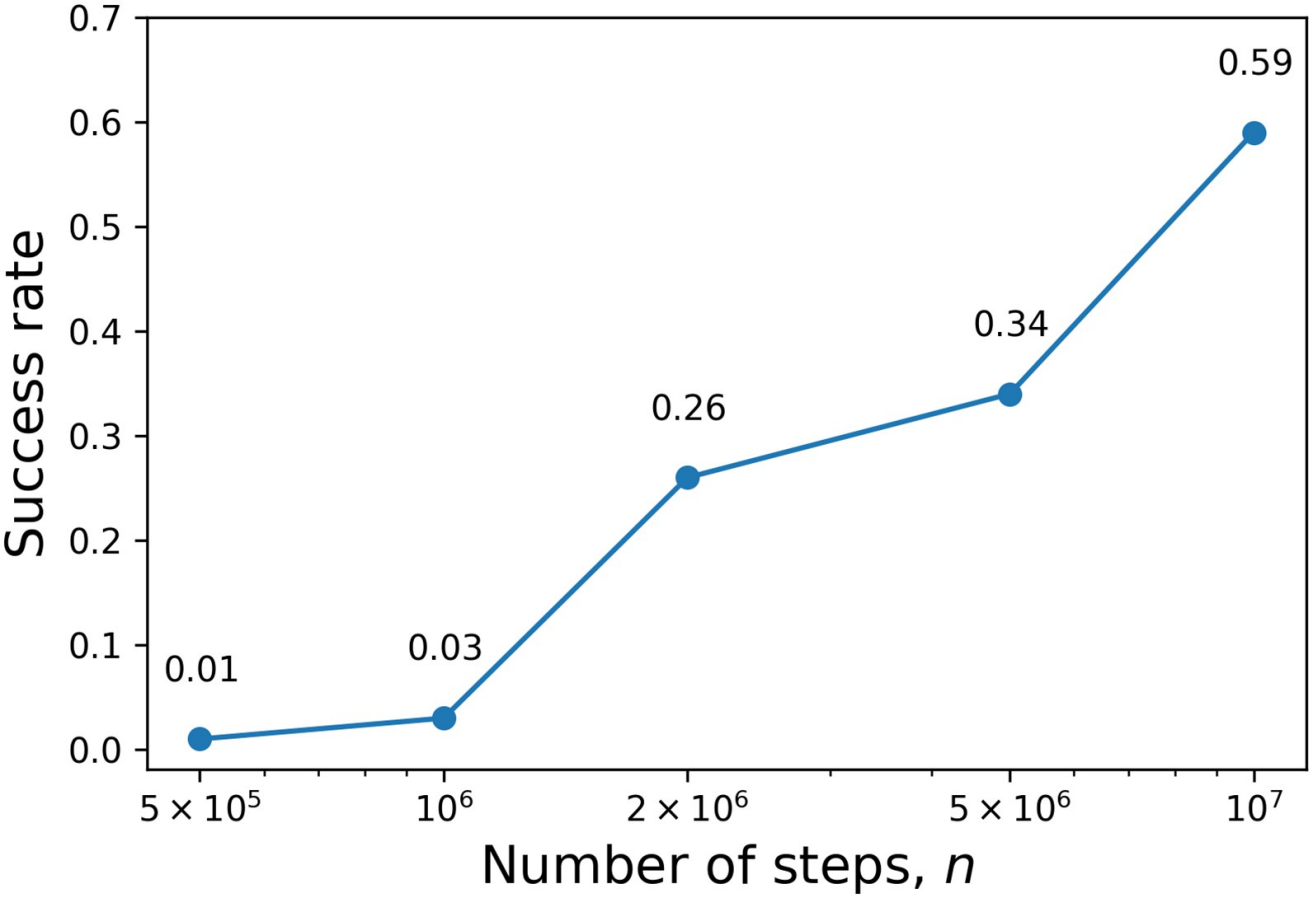
Success rate out of 100 trials in the SSA case.

Here $\Delta E_{i}=E_{{\underline{S}}^{\left(i\right)}}-E_{\underline{S}}$, and ${\underline{S}}^{\left(i\right)}$ is the same as $\underline{S}$ except the *i*th spin is flipped. If the initial temperature is *T*_init_ = 2max_*i*_ Δ*E*_*i*_, then any initial trial *i* will be accepted with more than 50%, so it can be considered a sufficiently high temperature. The final temperature is *T*_final_ = *T*_init_ *α*^*n*^. Since in the G-sets the *J*_*ij*_ elements are -1, 0 or 1, the smallest positive energy difference is 1, hence it is reasonable to choose *T*_final_ = 0.05. Below that temperature it is unlikely to accept any state with higher energy than the current one. The parameter of the geometrical schedule is $\alpha =\sqrt[{n}]{T_{\text{final}}/T_{\text{init}}}$. The only remaining hyperparameter is *n*. Fig 4 shows the success rate for different *n* values for G1. In 15 seconds, 10^6^ Monte Carlo steps can be carried out. It means if we use *n* = 2 × 10^6^, then we need approximately 115 seconds to find the best known Cut Value of G1.

For the B&B method, we used the BiqCrunch implementation [40]. In one hour, the Cut Value it could find was 11524.

The success rate can be dissimilar for the other sets. For example, for G3, after the hyperparameter optimization in the case of the QMFA, it is 0.016, and in the case of SSA with *n* = 5 × 10^7^, it is 0.17. Even the running time is increased to *t*_QMFA_ = (2.8 ± 1.8) seconds, resulting in the expected time for finding the best known Cut Value with QMFA to 175 seconds. With SSA, this expected time is 455 seconds, and the B&B method found a solution with the Cut Value of 11538 in one hour.

In the examples mentioned above, the QMFA outperformed both the SSA and the B&B. Still, for example, for the G2 problem, the QMFA was only able to find the best known Cut Value once out of 2000 trials hence it would be inconsistent to calculate the average success time. On the other hand, the SSA found 5 times the best Cut Value out of 100 trials with *n* = 5 × 10^6^ and the expected success time is 1500 seconds. The B&B method found a state with CV = 11521 in one hour.

The G11 is special since the QMFA cannot find the best Cut Value, the SSA can find it on an average of 2500 seconds, but the B&B method needs only 512 seconds. This can be due to its simple structure since the G11 is a toroidal graph. On the other hand, an additional one hour is not enough for B&B to confirm that it is the best possible Cut Value. Since it is a toroidal graph (in this case, an 8 × 100 grid with periodic boundaries), we can solve it exactly with the transfer matrix method, confirming that 564 is indeed the best possible Cut Value.

Considering all of the facts above, we can state that no algorithm is universally dominant, but we can say the QMFA can find a good, in some cases the best, optimum in a relatively short time.

## 6 Conclusion and outlook

We have shown that the thermal and the quantum mean-field annealing are governed by similar equations, where the temperature and the tunneling rate play similar roles. This is due to the similarity of the entropic term in the free energy and the transverse term in the energy of the quantum approach. The sigmoid function in the quantum approach differs from the thermal one, and for large arguments, it changes like a power law as opposed to the exponential in the thermal case. As it was pointed out in [29] the specific nonlinearity of the sigmoid function affects the speed of the convergence of the mean-field approach. This can be one of the reasons for the relative success of the present approach in reproducing some of the best cut values for certain problems from the G-set. The approximation presented here is the most straightforward application of the variational method. The next step can be a more sophisticated version of the mean-field annealing that can be introduced by considering the correlations among the most coupled spins, which might lead to even better cut values in the future.
